# Scientific Items

**Published:** 1884-01-20

**Authors:** 


### SCIENTIFIC ITEMS.

**Sugar from Rags.**—The following stale item is again on its rounds among our exchanges :

"Some years ago Mr. Pepper created some sensation by undertaking to make sugar from old shirts. Sugar is now manufactured in Germany from old rags. The rags are treated with sulphuric acid, and converted into dextrine. This is treated with a milk of lime, and is then subjected to a new bath of sulphuric acid, which converts it into glucose. The glucose obtained by this process is identical with that of commerce. and may be used in the same way for confections, ices, etc. When the manufacture has become more abundant, the price will doubtless be very small. It is known that a large number of substances are capable of transformation into glucose. The cellulose or fibrous tissue of wood, treated with sulphuric acid, is changed into dextrine and glucose, and glucose is industrially produced from starch."

Glucose was made from linen rags by Braconnot in 1819 (Annales Chim. Phys. [2], xii., 181), hence this process can hardly lay claim to novelty. We also doubt the truth of the assertion that sugar is *now* made from rags in Germany, for two reasons. In the first place potatoes are very abundant there, and potato starch is far cheaper than rags ; in the second place the rags will sell for more to paper makers than the glucose would bring when finished. It is true that careless chemists frequently convert their shirts and aprons accidentally into glucose by spilling sulphuric acid on them, but the process is no more economical than making picric acid by the action of nitric acid upon the human fingers.—*Drug. Cir. & Chem. Gaz.*

**Terra Cotta Lumber.**—There is a "terra cotta lumber company" at Perth Amboy, New Jersey. The World describes the visit of some scientists and others to the works, and states that the product is a very useful article. Terra cotta lumber is made by mixing kaolinite or "top" clay with saw-dust until the consistency of dough is obtained, when it is cast in large square blocks and burned in kilns in the same manner as ordinary brick. The "lumber" has no fibrous texture like wood, "the strength of the material arising from incipient vitrifaction" obtained in firing it. Half-inch boards, smoothly planed and jointed, "show greater strength and tenacity than dry oak of equal thickness." Every shape which can be given to wood by edged tools can be given to terra cotta lumber. It is as easily worked as pine or spruce, is half the weight of building brick, and retains plaster without the aid of lathing. The adaptability of the new material for building purposes is said to be excellent in other ways.—*Industrial Gazette.*

**Styrian Steel** is the name of a new brand, which has recently been put on the market and is gaining many friends. It is made in Styria, of special ores, and shows extraordinary endurance.—*Ex.*

**Photographic Progress.**—Photography appears to be running a race with electricity in curious developments and novel applications. A New York photographer has just achieved the remarkable feat of photographing sound waves instantaneously. The instrument upon which the sound waves were made visible was a new telephone, the diaphragm upon which the voice is projected having a fine metallic point mounted on the centre of its reverse side. This point met the pointed end of a conducting wire so nearly that when at rest the interval between the two points was discovered only through a strong lens. The thing to be done was to show, in a series of pictures, the alternate contact and separation of the points from the vibrations imparted to the diaphragm by the voice, involving the closing and opening of the electrical circuit and the consequent reproduction of the same rate of vibration in the receiving instrument at the other end of the line. The photographer succeeded in this, the plates taking an impression of each electric spark that resulted when the points touched each other in the vibration of the diaphragm. The photographs printed from them showed under the glass, in some, contact of the points, and in others, a variety of infinitesimally differenced intervals between them. Not one of the impressions had more than one twenty-four thousandth of a second in which to be begun and ended, according to Wheatstone's investigations.—*Industrial Gaz.*

**New Electric Power.**—The most astonishing claim yet made in behalf of electricity is that it has been proven possible to convey by it vibrations of light, so that it is practicable not only to speak with a distant friend but to see him. According to the Otago Times, Dr. Guidrah, of Victoria, has invented an apparatus called by him the electroscope, which accomplishes this. The paper in question says that a public test of this instrument was made in Melbourne, in the presence of some forty scientific and public men. "Sitting in a dark room, they saw projected on a large disc of white burnished metal the race course at Flemington, with its myriad hosts of active beings. Each minute detail stood out with perfect fidelity to the original, and as they looked at the wonderful picture through binocular glasses, it was difficult to imagine that they were not actually on the course itself, and moving among those whose actions they could so completely scan."—*Ex.*

**A Plastic mass,** which is valuable for many purposes, is made under a recent German patent, by well mixing, in equal proportions, ground calcined and paste magnetite, and a solution of sulphate of magnesia of 1.9 specific gravity, and running the mixture into oiled molds. The mass can be washed with warm water and soap, when hardened, and will have the appearance of white marble, the hardness of which it also acquires with time. This new substance is called "Marmorin."—*Ex.*

THE first cotton mill in California is soon to be built at Oakland. The southern part of the State is regarded as favorable to cotton culture.—*Mechanical News.*

